# Age-related changes in geometry and transparency of human crystalline lens revealed by optical signal discontinuity zones in swept-source OCT images

**DOI:** 10.1186/s40662-023-00365-y

**Published:** 2023-12-01

**Authors:** Ashish Gupta, Daniel Ruminski, Alfonso Jimenez Villar, Raúl Duarte Toledo, Grzegorz Gondek, Barbara Pierscionek, Pablo Artal, Ireneusz Grulkowski

**Affiliations:** 1 Institute of Physics, Faculty of Physics, Astronomy and Informatics, Nicolaus Copernicus University in Toruń, Grudziądzka 5, 87-100, Toruń, Poland; 2 Laboratorio de Óptica, Centro de Investigación en Óptica y Nanofísica, Universidad de Murcia, Edif. CIOyN, N º34, Campus de Espinardo, 30100 Murcia, Spain; 3 Faculty of Health, Education, Medicine, and Social Care, Medical Technology Research Center, Chelmsford Campus, Anglia Ruskin University, Bishop Hall Ln, Chelmsford, CM1 1SQ UK

**Keywords:** Crystalline lens, Ageing, Optical signal discontinuity zones, Optical coherence tomography

## Abstract

**Background:**

The shape and microstructure of the human crystalline lens alter with ageing, and this has an effect on the optical properties of the eye. The aim of this study was to characterise the age-related differences in the morphology and transparency of the eye lenses of healthy subjects through the optical signal discontinuity (OSD) zones in optical coherence tomography (OCT) images. We also investigated the association of those changes with the optical quality of the eye and visual function.

**Methods:**

OCT images of the anterior segment of 49 eyes of subjects (9–78 years) were acquired, and the OSD zones (nucleus, C1–C4 cortical zones) were identified. Central thickness, curvature and optical density were measured. The eye’s optical quality was evaluated by the objective scatter index (OSI). Contrast sensitivity and visual acuity tests were performed. The correlation between extracted parameters and age was assessed.

**Results:**

The increase in lens thickness with age was dominated by the thickening of the cortical zone C3 (0.0146 mm/year). The curvature radii of the anterior lens surface and both anterior and posterior nucleo-cortical interfaces decreased with age (− 0.053 mm/year, − 0.013 mm/year and − 0.006 mm/year, respectively), and no change was observed for the posterior lens radius. OCT-based densitometry revealed significant correlations with age for all zones except for C1β, and the highest increase in density was in the C2–C4 zones (R = 0.45, 0.74, 0.56, respectively, *P* < 0.001). Increase in OSI was associated with the degradation of visual function.

**Conclusions:**

OCT enables the identification of OSD zones of the crystalline lens. The most significant age-related changes occur in the C3 zone as it thickens with age at a faster rate and becomes more opaque than other OSD zones. The changes are associated with optical quality deterioration and reduction of visual performance. These findings contribute to a better understanding of the structure–function relationship of the ageing lens and offer insights into both pathological and aging alterations.

**Supplementary Information:**

The online version contains supplementary material available at 10.1186/s40662-023-00365-y.

## Background

The human crystalline lens is a biconvex adjustable refractive element of the eye and consists of concentric layers of fibre cells that form the lens nucleus and cortex. Up to the sixth decade of life, the lens can change its shape to allow for focus on a range of distances. This functional process, called accommodation, gradually depletes with age, rendering the older eye unable to focus on near objects without spectacle correction (presbyopia) [1]. The lens is subject to ageing processes that alter its structural, optical and biomechanical properties [2, 3]. Lens transparency requires specific structural organisation [3, 4]. Age-related structural changes that lead to increased intraocular light scattering can manifest as opacification and a deterioration in vision [2, 3]. Accordingly, assessing age-related features may provide insights into structural and functional changes and improved understanding of age-related vision conditions such as presbyopia and cataract [5].

**Fig. 1:**
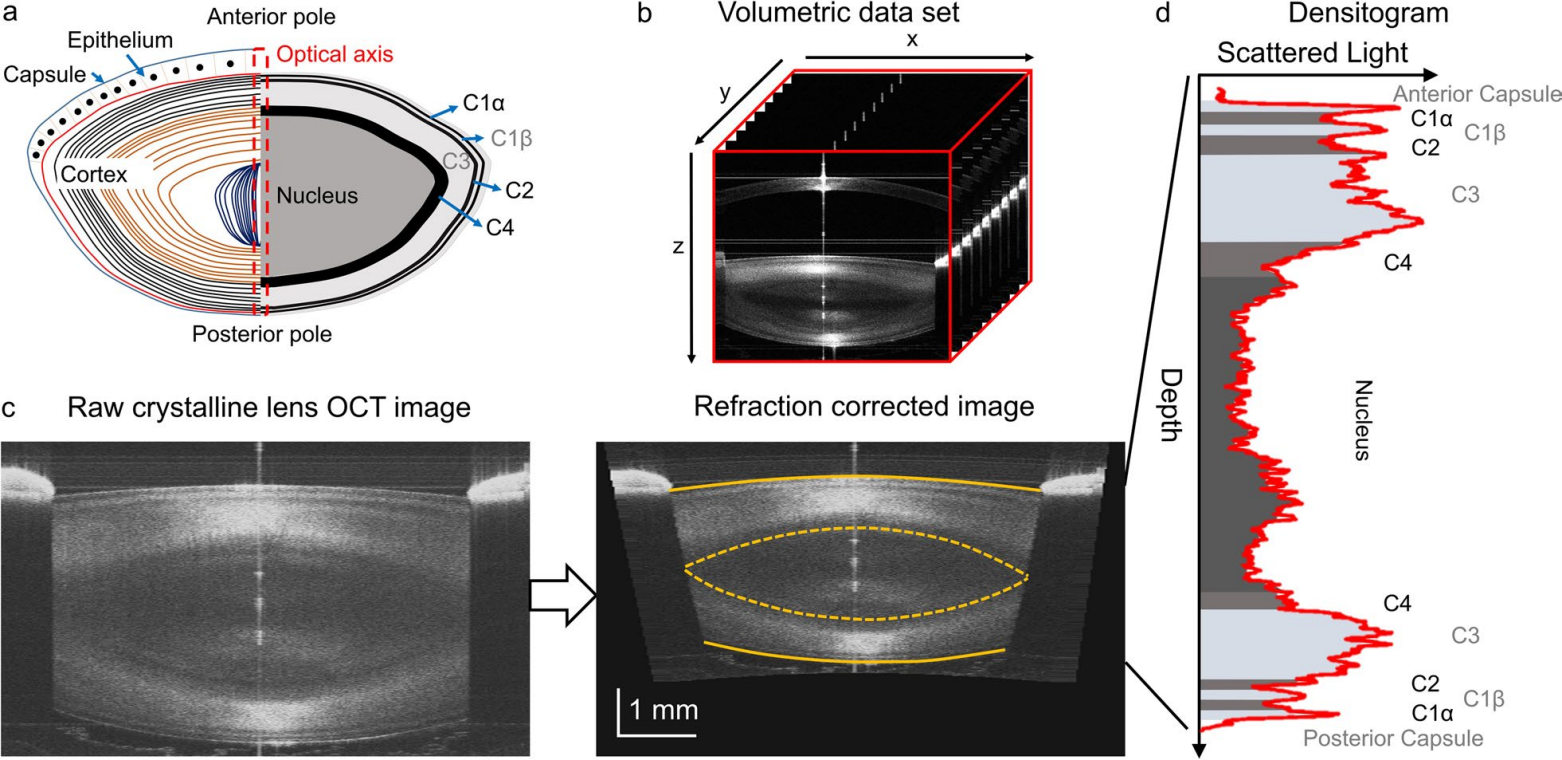
OCT data processing. **a** Schematic diagram of the crystalline lens [left half: structure of the crystalline lens, and right half: optical signal discontinuity (OSD) zones] and region of interest (ROI, red) for generating densitograms for the zones. The epithelial cell layer is not shown to scale. **b** Three-dimensional swept-source OCT volumetric (8 × 8 × 7 mm^3^) dataset of a 49-year-old subject. **c** Refraction correction of the central B-scan. The surfaces of the anterior and posterior of the lens (solid yellow) and the nucleus (dashed yellow) were used to measure the radii of curvature. **d** Densitogram of the lens and identification of the OSD zones based on Oxford system nomenclature. Hyper-reflective zones are indicated by bright grey colour. Hypo-reflective zones are indicated by dark grey colour. The thickness and optical density were measured for each zone

In vivo and in vitro age-related studies revealed changes in human lens morphology, light transmission, gradient refractive index distribution and biomechanics using different imaging modalities [6–15]. However, most clinical devices visualising ocular structures are based on the detection of light scattering that occurs at optical inhomogeneities (microscopic refractive index variations) within ocular structures. In particular, Scheimpflug photography was initially utilised to extract quantitative information about the changes in lens shape and densitometry with age [6, 10–12, 16]. Using biomicroscopic or Scheimpflug imaging, characteristic light and dark regions are seen in transparent lenses and have been referred to as optical signal discontinuity (OSD) zones or the zones of discontinuity [2, 3, 10, 17]. Observation of OSD zones can be explained by the spatial modulation of the gradient index profile within the lens, and it can be linked to the lens development and growth [18, 19]. The OSD zones in slit lamp and Scheimpflug images have been used for the Oxford Cataract Classification and Grading System that is based on the identification of four cortical zones (C1, C2, C3 and C4) and the nucleus (Fig. 1a) [20].

Optical coherence tomography (OCT) enables the mapping of back-reflected/back-scattered light and generation of cross-sectional and three-dimensional images of the eye [21, 22]. OCT was used to characterise human lens ageing by measuring the shape and refractive status in vivo and in donor eyes [23–31]. In addition, in vivo OCT imaging of the lens was used to identify and quantify opacities in different types and grades of cataracts and to visualise lens sutures [32–34]. However, the features of internal lens structures, including OSD zones, have not been analysed from OCT images.

Therefore, the aim of this study was to characterise the age-related differences in the OSD zones of the lens. Micrometre-scale resolution and high sensitivity, i.e., the ability to detect low signals, are the main advantages of OCT with respect to other clinically used imaging methods that facilitated the precise identification of OSD zones. Volumetric images of the lens acquired with a bench-top swept-source OCT system were used to measure the geometrical dimensions and optical density of the human lens zones. In addition, the association of those parameters with the optical quality of the eye and with visual function was investigated.

## Methods

### Data acquisition

This cross-sectional study was undertaken by recruiting 50 participants (with ages ranging from 9 to 78 years) to the Laboratory of Optics at the University of Murcia. The study adhered to the Tenets of the Declaration of Helsinki. Participants were informed about the nature of the study, and written consent was obtained from each participant and in the case of children, parental consent was also obtained. The Institutional Review Boards at Nicolaus Copernicus University (KB290/2019) and the University of Murcia (3.10.2013) approved the study. Standard ophthalmic examination was conducted for all participants; individuals with previous ocular surgery were excluded from the study. All eyes were imaged in the unaccommodated state and with no pupil dilation. The measurements were performed in mesopic conditions. One participant was rejected because of large motion artifacts that degraded OCT image quality. One randomly selected eye from each participant was taken for statistical analysis to avoid correlations between fellow eyes. Accordingly, the data from 49 eyes (25 males and 24 females) were included in the study.

A custom-made swept-source OCT instrument was used with a wavelength sweeping laser centered at 1.05 μm and a sweep rate of 50 kHz [33]. The performance of the prototype system was optimized to feature anterior segment imaging (including the entire lens) by: selection of coherence properties of the light source and the bandwidth of the detector and acquisition card, minimization of the signal drop with depth, the design of the illumination optics (light delivery system), adjustment of focal plane with respect to zero-delay line, and scanning strategy equivalent to Enhanced Depth Imaging in retinal (choroidal) imaging [33]. The OCT system sensitivity was 103 dB, and it had a reduced sensitivity drop over the entire imaging depth of 22.2 mm in the air, enabling imaging of the entire depth of the human anterior segment with an axial resolution of 8 μm and lateral resolution of 43 μm. Three-dimensional volumetric data consisting of 300 A-scans × 300 B-scans were acquired by raster scanning of the central area of 8 × 8 mm^2^ of the eye.

The parameters of the OCT instrument, especially sensitivity, were regularly checked using a standard procedure. Moreover, the eye was always carefully aligned with respect to the scanning head by an experienced operator to assure that the signal from the anterior segment was always properly positioned with respect to the zero-delay line and the center of the scan area. This allowed for an optimized OCT image quality for each eye and provided consistent imaging conditions for the whole cohort, which in turn resulted in more objective quantitative analysis of the data.

A double-pass technique-based optical quality analysis system (OQAS, HD Analyzer II, Visiometrics SL, Terrassa, Spain) was used to evaluate the optical quality of the eye objectively and to determine the objective scatter index (OSI) [35, 36]. The visual function was assessed with an adaptive optics visual simulator (VAO; Voptica SL, Murcia, Spain) [37–41]. High-definition display technology in VAO allowed presentation of stimuli for visual acuity (VA) measurement and contrast sensitivity testing and determination of the area under the log contrast sensitivity function (AULCSF). VAO was also used to measure subjective refraction.

The measurements were performed in the following order: contrast sensitivity test, subjective refraction, VA (with VAO), OSI (with OQAS) and OCT.

### OCT data post-processing

Volumetric data were represented by a series of cross-sectional images (Fig. 1b). Each three-dimensional data set (x–y–z, 300 × 300 × 1500 voxels, voxel size: 26.7 × 26.7 × 8.9 μm^3^ in air) was corrected for motion artefacts. To correct the OCT images for light refraction, the corneal and lenticular interfaces were segmented by fitting these with 2nd order polynomials. Hence, a double-shell model was assumed to simplify the gradient index lens in a ray-tracing refraction correction algorithm. The following refractive indices were taken to calculate light propagation through air—cornea—aqueous humour—anterior cortex—nucleus—posterior cortex—vitreous humour: 1, 1.3665, 1.3266, 1.3749, 1.3947, 1.3749, and 1.3270, respectively [38]. The OCT images, corrected for light refraction, allowed measurement of the morphological features (radii of curvature and central thicknesses). Radii of curvature were determined within the 3.5 mm-diameter region (Fig. 1c). Optical density of the lens nucleus, cortex or the whole lens was calculated by averaging the signal in OCT image in the corresponding part of the lens. The approach is similar to clinical Scheimpflug imaging densitometry and other OCT-related studies [42–45]. Higher OCT signal (brighter images) correspond to optically denser lens.

The region of interest (ROI) was selected subsequently, spanning a radius of 66.5 µm around the central axis connecting anterior and posterior lens apices. Hence, the ROI had the shape of a cylinder spreading from the anterior to the posterior lens (Fig. 1a, b). Data within the ROI were averaged laterally, and a densitogram was generated. The size of the ROI was selected to avoid the influence of the curvature of the lens surface on the densitometric profile. The speckle noise was removed from the densitogram by smoothing with the five-pixel moving window. The OCT-based densitograms were used to identify the OSD zones according to the Oxford nomenclature: C1α, C1β, C2, C3, C4, and the nucleus (Fig. 1d) [17, 20]. The locations of the zone boundaries were obtained manually by a single grader using the inflection point positions (maxima of the gradient) found between the peaks and valleys in the densitometric curve [17]. The segmentation of OSD zones was the only manual step in the OCT data post-processing. The thickness and optical density within each OSD zone were also measured.

### Data analysis

Statistical analyses were completed using Microsoft Office Professional Plus Excel 2013 (Microsoft Corp., Redmond, WA, USA). Intra-observer reproducibility and inter-observer reproducibility for the thicknesses and optical densities of the OSD zones were assessed by calculating intraclass correlation coefficients (ICC) (see also Additional file 1). Spearman’s rank correlation was performed to investigate age-related changes in the lenticular morphology and in lens transparency. Statistical significance of Spearman’s correlation coefficient (R_s_) was tested at the significance level α = 0.05. False discovery rate control in the main correlation analysis was obtained by applying the Benjamini–Hochberg procedure (R v.4.3.0). Correlation coefficients were also generated to observe associations between parameters extracted from OCT, OQAS and VAO, and to investigate the impact of intraocular scattering on vision. Further analysis was performed by categorising the extracted data into three age groups (Group 1: 9 to 29 years, Group 2: 30 to 54 years, and Group 3: 55 to 78 years) to observe the percentage contribution of OSD zone thickness to the growth in lens thickness. The differences of relative OSD thickness between the groups were tested with the Mann–Whitney U test.

Influencing factors on lens thickness and optical density were also explored with multivariable linear regression model (MVLR) analysis. The predictors for both dependent parameters were checked for homoscedasticity using the Breusch–Pagan test. The multicollinearity was checked by computing the variance inflation factor; variables with high multicollinearity were excluded to keep the analysis unbiased.

## Results

**Fig. 2:**
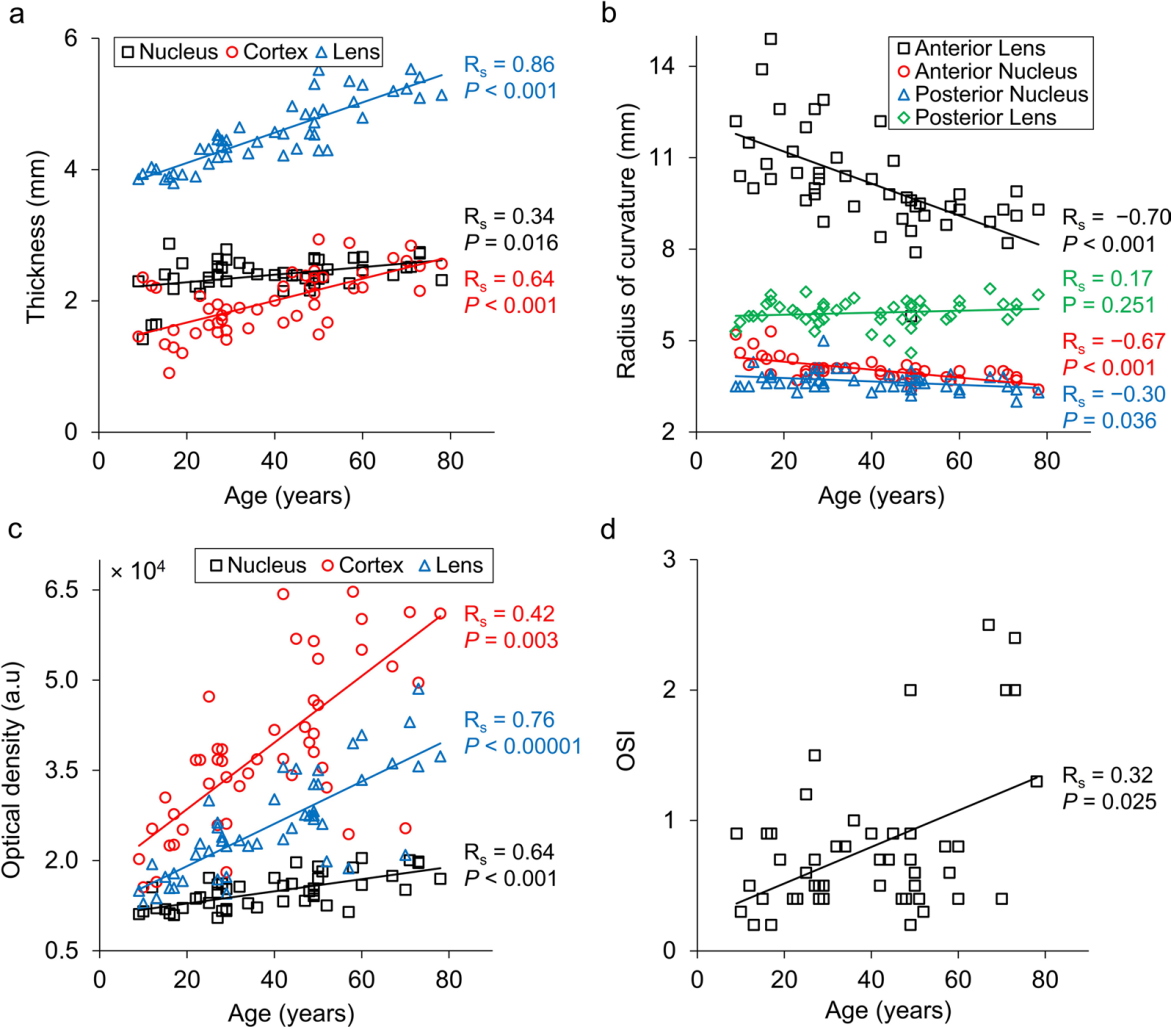
Age dependence of measured (**a**) thickness of nucleus, cortex, and whole lens, (**b**) radii of curvature of the anterior and posterior lens capsules and the lens nucleus, (**c**) optical density of the nucleus, cortex, and lens, and (**d**) objective scatter index (OSI). All eyes were unaccommodated

The characteristics of the study group are given in the Table 1. Mean diameter of the pupil was 5.8 ± 1.4 mm, and age-related decrease in pupil size was observed (R_s_ = − 0.62, *P* < 0.001). Thus, the quantitative image analyses were restricted to the minimum pupil size of 3.5 mm. High reproducibility was found for all parameters extracted from OCT except for the thickness of the C4 zone (see also Additional file 1). Morphological and densitometric parameters of the lens derived from OCT data, OSI, VA and AULCSF were correlated with age. The results are summarised in Table 2, and selected plots are presented in Fig. 2. The central thickness of the lens and identified lens layers increased with age with high and moderate correlations (R_s_ = 0.34, 0.64, and 0.86 for the nucleus, cortex, and the whole lens, respectively; Fig. 2a). As overall lens thickness grows at the rate of 0.023 mm/year, the cortex thickens at 0.017 mm/year and the nucleus at 0.006 mm/year (Table 2). Among all cortical OSD zones, the highest correlation between thickness and age was found for the C3 layer (R_s_ = 0.70, *P* < 0.001), and the thickening rate of the C3 zone (0.0146 mm/year) was close to that of the entire cortex. The thickness of the C1α zone also correlated significantly with age. Conversely, the changes in the thicknesses of C1β, C2 and C4 do not show a significant correlation with age.

**Table 1:** Characteristics of the study group and average parameters of morphology and transparency of the lens and vision performance

Parameter	N	Mean ± SD
Age (years)	49	39.24 ± 18.80
Subjective refraction error (D)	49	
Sphere		− 1.0 ± 2.2
Cylinder		− 0.6 ± 0.6
Pupil diameter (mm)	49	5.8 ± 1.4
Thickness (mm)	49	
Lens		4.54 ± 0.5
Cortex		1.99 ± 0.47
Nucleus		2.39 ± 0.27
C1α		0.16 ± 0.04
C1β		0.20 ± 0.07
C2		0.27 ± 0.08
C3		1.07 ± 0.38
C4		0.30 ± 0.08
Radius of curvature (mm)	49	
Anterior lens capsule		10.19 ± 1.63
Anterior nucleus		4.06 ± 0.37
Posterior nucleus		3.67 ± 0.33
Posterior lens capsule		5.91 ± 0.40
Optical density (× 10^3^) (a.u.)	49	
Lens		25.8 ± 8.5
Cortex		35.0 ± 6.5
Nucleus		14.8 ± 2.9
C1α		33.6 ± 7.4
C1β		39.8 ± 7.4
C2		33.1 ± 8.9
C3		4.3 ± 1.1
C4		25.5 ± 6.5
Objective scatter index	49	0.75 ± 0.55
Visual function	49	
AULCSF		2.25 ± 0.20
VA (logMAR)		− 0.01 ± 0.06

*SD* = standard deviation; *AULCSF* = area under the log contrast sensitivity function; *VA* = visual acuity

**Table 2:** Spearman’s correlation of measured parameters with age

Dependent variable	R_s_	*P* value	FDR-corrected *P* value	Rate of change (per year)
Thickness (mm)				
Lens	0.86*	< 0.001	< 0.001	0.023
Cortex	0.64*	< 0.001	< 0.001	0.017
Nucleus	0.34*	0.016	0.024	0.006
C1α	0.41*	0.003	0.007	0.001
C1β	0.25	0.083	0.101	0.0011
C2	− 0.02	0.892	0.892	− 0.0002
C3	0.70*	< 0.001	< 0.001	0.0146
C4	0.1	0.494	0.541	0.0006
Radius of curvature (mm)				
Anterior lens capsule	− 0.70*	< 0.001	< 0.001	− 0.053
Anterior nucleus	− 0.67*	< 0.001	< 0.001	− 0.013
Posterior nucleus	− 0.30*	0.036	0.046	− 0.006
Posterior lens capsule	0.17	0.242	0.279	0.003
Optical density (a.u.)				
Lens	0.76*	< 0.001	< 0.001	351
Cortex	0.42*	0.003	0.006	552
Nucleus	0.64*	< 0.001	< 0.001	101
C1α	− 0.37*	0.009	0.014	− 128
C1β	− 0.05	0.733	0.766	− 33
C2	0.45*	0.001	0.003	234
C3	0.74*	< 0.001	< 0.001	434
C4	0.56*	< 0.001	< 0.001	200
Objective scatter index	0.32*	0.025	0.034	0.014
Visual function				
AULCSF	− 0.38*	0.007	0.013	0.0047
VA (logMAR)	0.37*	0.008	0.014	0.0005

*FDR* = false discovery rate; *AULCSF* = area under the log contrast sensitivity function; *VA* = visual acuity

Significance of Spearman’s correlation coefficient R_s_ was indicated with an asterisk

The radii of curvature of the anterior lens capsule and both nuclear interfaces declined with age (coefficients R_s_ = − 0.70, − 0.67, and − 0.30, respectively), and statistical significance of the correlation was found (Fig. 2b). A substantial decrease in radius of curvature of the anterior lens was observed according to this equation: anterior lens radius [mm] = − 0.053(± 0.011)*Age [years] + 12.25(± 0.45) (Table 2). Although the posterior lens capsule became slightly flatter as described by this equation: posterior lens radius [mm] = 0.0032(± 0.0031)*Age [years] + 5.787(± 0.004), no statistically significant correlation between its radius of curvature and age was found (R_s_ = 0.17, *P* = 0.304).

Light scattering by the lens was expressed by the optical density defined as the average OCT signal taken from a particular region of the lens. The optical density of the whole lens, the cortex and the nucleus increased significantly with age (with R_s_ = 0.76, 0.42, and 0.64, respectively; Fig. 2c). Unlike zone C1, all cortical OSD zones demonstrated increased back-scatter with age (Table 2). A similar trend was observed for intraocular forward scatter, but the correlation of OSI with age was lower (R_s_ = 0.32, *P* = 0.025; Fig. 2d). Vision quality degradation with age was characterised by a statistically significant decrease in AULCSF (R_s_ = − 0.38, *P* = 0.007) and an increase in VA that was expressed in logMAR units (R_s_ = 0.37, *P* = 0.008).

The correlation of parameters extracted from OCT with optical quality of the eye and vision are shown in Table 3 (see also Additional file 2). The optical density of the lens was negatively correlated with AULCSF (R_s_ = − 0.35, *P* = 0.0126), but no correlation was found with VA (R_s_ = 0.23, *P* = 0.1080). Additionally, OSI showed statistically significant correlations with parameters describing the degradation of vision (R_s_ = − 0.42, *P* = 0.0027 for AULCSF and R_s_ = 0.40, *P* = 0.0045 for VA) and the increase in back-scatter in the lens resulted in an increase in intraocular scatter expressed by OSI (R_s_ = 0.41, *P* = 0.0036). The correlation coefficients between lens optical density, OSI, AULCSF and VA, however, indicate that these have weak relationships.

**Table 3:** Spearman’s correlation between the measurements from optical coherence tomography (OCT), optical quality analysis system (OQAS), objective scatter index (OSI), visual adaptive optics simulator (VAO)

Parameter	Lens optical density (a.u.)	OSI	AULCSF	VA (logMAR)
**Lens optical density (a.u.)**				
**OSI**	R_s_ = 0.41* (*P* = 0.0036)			
**AULCSF**	−0.35* (*P* = 0.0126)	−0.42* (*P* = 0.0027)		
**VA (logMAR)**	0.23 (*P* = 0.1080)	0.40* (*P* = 0.0045)	−0.60* (*P* < 0.0001)	

Significance of Spearman’s correlation coefficient R_s_ was indicated with an asterisk. Weak correlations were found between lens optical density, OSI, AULCSF and VA

*OSI* = objective scatter index; *AULCSF* = area under the log contrast sensitivity function; *VA* = visual acuity

**Fig. 3:**
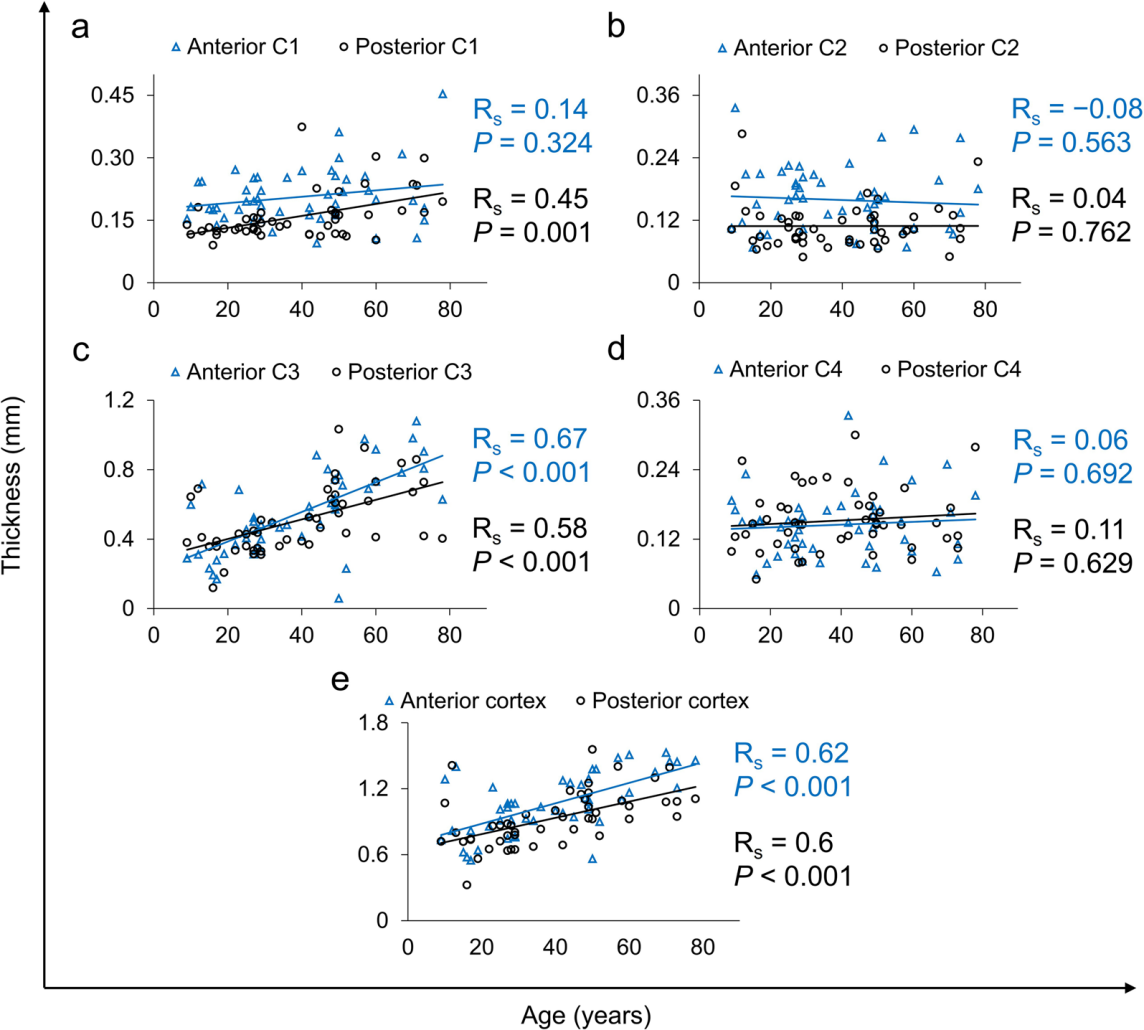
Correlation of ageing with changes in the anterior and posterior thickness of the cortical optical signal discontinuity (OSD) zones: (**a**) C1, (**b**) C2, (**c**) C3, (**d**) C4 and (**e**) the cortex. No significant differences between the growth rates of corresponding anterior and posterior OSD zones were detected

**Fig. 4:**
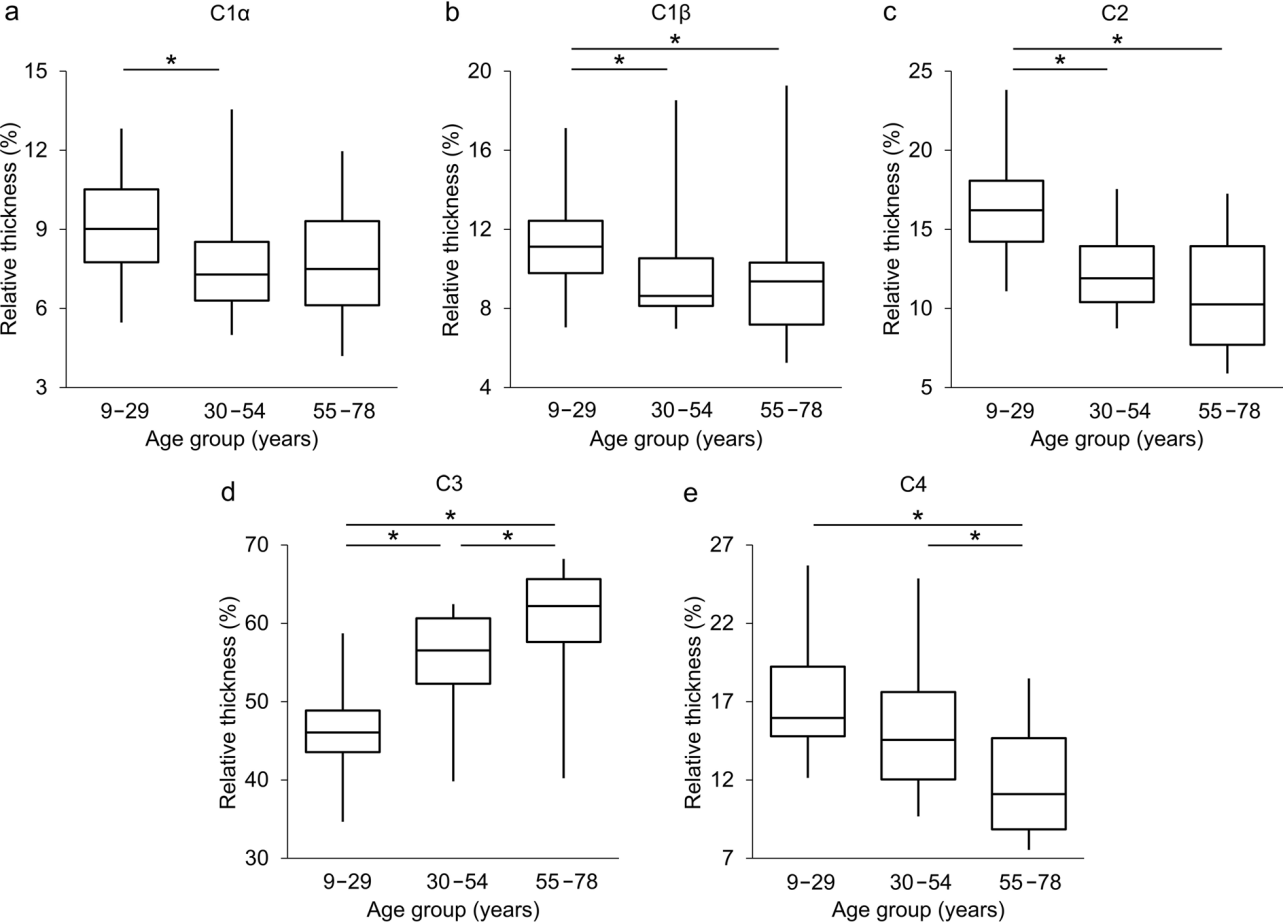
Percentage contribution of cortical layers to the total cortical thickness for different age groups: (**a**) C1α, (**b**) C1β, (**c**) C2, (**d**) C3, (**e**) C4. Box plots show the interquartile ranges, horizontal lines indicate the median. * indicates statistical significance

A more detailed analysis was completed for the thickness of the lens and each OSD zone. No significant differences between the increase in the rate of thickness of corresponding anterior and posterior OSD zones were found (Fig. 3). The data were further investigated by categorisation based on age groups. The entire population was split into three age groups (Group 1: n = 21, mean age 21.3 ± 6.9 years; Group 2: n = 18, mean age 44.9 ± 6.1 years; Group 3: n = 10, mean age 66.7 ± 7.5 years). In Fig. 4, box plots depict the contribution of each cortical zone to the overall cortex thickness in each subject. Statistical significance between the groups is given in Table 4. The bright C3 zone was mainly responsible for the growth of the overall cortex, and thus contributed to the development of the crystalline lens (from 46% to 62% in Fig. 4c). Furthermore, relative thickness of the zones C1β, C2 and C4 decreased with age (Fig. 4b, d, e).

**Table 4:** Statistical significance (*P* values) in relative thickness of the optical signal discontinuity (OSD) zones between age-groups assessed with the Mann–Whitney U test

OSD zone	Group 1 *vs.* Group 2	Group 2 *vs.* Group 3	Group 1 *vs.* Group 3
C1α	0.0414*	0.9840	0.1336
C1β	0.0232*	0.8259	0.0366*
C2	0.0003*	0.2585	0.0026*
C3	0.0005*	0.0366*	0.0005*
C4	0.2420	0.0466*	0.0022*

Group 1 represented subjects aged 9 to 29 years, Group 2 represented subjects aged 30 to 54 years, and Group 3 represented subjects aged 55–78 years. Statistical significance is indicated by an asterisk

MVLR analysis showed that the thickness of the nucleus (β = 0.27, *P* < 0.001) and cortical layers (C1α, β = 0.04, *P* = 0.021; C1β, β = 0.07, *P* = 0.001; C2, β = 0.1, *P* < 0.001; C3, β = 0.38, *P* < 0.001; C4 β = 0.09, *P* < 0.001) had a strong influence on the overall lens thickness, with adjusted R^2^ = 0.97. Lens optical density was highly influenced (adjusted R^2^ = 0.87) by optical densities of the nucleus (β = 4835, *P* < 0.001) and the zone C3 (β = 2997, *P* = 0.04) as well as by OSI (β = 2025, *P* = 0.004). However, no significant influence (*P* > 0.05) of optical densities of C1α (β = − 1029), C1β (β = − 466), C2 (β = 1197), C4 (β = − 236), AULCSF (β = 102) or VA (β = 456) were detected. MVLR also revealed that VA was influenced by AULCSF (β = − 0.001, *P* = 0.007), the radius of curvature of the anterior lens (β = 0.022, *P* = 0.025) and the radii of anterior and posterior nuclei (β = − 0.019, *P* = 0.0098 and β = 0.015, *P* = 0.042, respectively).

## Discussion

In this cross-sectional study, a detailed quantitative analysis of age-related alterations of morphology and transparency of the human lens was undertaken and correlated with changes in visual function. Previous studies, using Scheimpflug imaging, identified OSD zones that are thought to be a consequence of optical inhomogeneity in the structure of the crystalline lens [18]. However, the limited resolution of Scheimpflug images made it impossible to divide the C1 zone into C1α and C1β. Furthermore, a clear boundary between the C4 zone and the nucleus could not be distinguished, resulting in C4 being excluded from the measurements [17]. We took advantage of specific features offered by swept-source OCT modality, such as high resolution and sensitivity that enabled acquisition of high-quality images and allowed for a new application of OCT in the identification of OSD zones in densitograms. Although it was possible to identify the C4 nuclear boundary in OCT, the manual segmentation of that zone was challenging for the observer, as indicated by our reproducibility study (see also Additional file 1). In principle, OCT outperforms Scheimpflug imaging in terms of resolution and in the capacity to detect low back-scattered signals, permitting more details to be extracted and quantified [46, 47]. The instrument used in this study was optimized to enable assessment of light back-scattering in the lens, and the inclusion criteria ensured that no corneal opacifications were evident in any of the eyes studied ensuring no degradation of OCT image quality.

### Lens thickness

This study confirmed that the central lens thickness increases with age, and the slope of age dependence of the lens thickness was estimated to be 0.023 mm/year, which is within the range of 0.019–0.029 mm/year observed with Scheimpflug photography, magnetic resonance imaging and OCT [9–12, 16, 24, 31, 48–51]. The increase in lens thickness with age is mainly governed by the thickening of the cortex, which grows at a rate three times faster than that of the nucleus (0.017 mm/year vs. 0.006 mm/year, Fig. 2a). The differences between the rate of thickness increase could also be observed within the cortical OSD zones. The thickness of the C1α and C3 zones increased with age significantly (0.0010 mm/year and 0.0146 mm/year, respectively). This is contrary to the findings by Dubbelman et al. who demonstrated that only the C2 zone had a dominant and statistically significant role in lens thickening with age, with a higher rate of age-related thickening in the anterior cortex [17]. Unlike Dubbelman et al. and Cook et al., we did not detect any significant differences in thickness change rates between the anterior and posterior cortices (Fig. 3) [6, 17]. These discrepancies could be explained by fundamental technological differences between Scheimpflug imaging and OCT. Although both modalities detect scattered light, they operate at different wavelengths and acquire signals at different scattering angles. Consequently, the densitometric profiles extracted from both imaging methods may present variations, which can impact C2 and C3 zone segmentation (although segmentation criteria are the same). The other factors influencing the results are the poorer resolution of the Scheimpflug camera and the low accuracy of determination of all OSD zones from Scheimpflug images in the previous studies [6, 17].

Furthermore, the mutual contribution of each OSD zone to the overall thickness of the cortex was investigated. The comparison within age groups revealed that the contribution of the C3 zone increased from 46% to 62% of the total thickness at the expense of the C1β, C2 and C4 zones (Fig. 4b–e). Therefore, it appears that all hyper-reflective zones of the OSD in the crystalline lens expand in size with age. In contrast, hypo-reflective zones C2 and C4 do not show a consistent increase in thickness, and this may be that their rate of growth is relatively slower than other zones, that these zones do not increase in thickness or that there may be some tissue compaction in these zones. Whether or not compaction occurs in the lens is not conclusive and appears to depend on species [52]; the nature of such a force has never been elucidated.

### Radii of curvature

The results showed a statistically significant decrease in the anterior lens radius, anterior nucleus radius and posterior nucleus radius at the rates of − 0.053 mm/year, − 0.013 mm/year and − 0.006 mm/year, respectively. However, no significant ageing trend in the posterior lens radius was observed (Fig. 2b). Our data suggest an agreement with the conclusions from previous in vivo studies where the anterior lens surface became significantly steeper (decreasing in rate between − 0.076 and − 0.044 mm/year), whilst the relation between posterior lens radius and age was either insignificant or marginally significant [7, 11, 16, 28, 29, 50]. The regression analysis of age dependence of anterior lens radius also showed good agreement of slope (rate) and intercept with corresponding relations obtained in other studies [7, 11, 16, 28, 29, 50]. However, the age-related differences of the curvature of nucleo-cortical interfaces are not available in the literature although lens modelling suggests that nuclear curvature becomes flatter with age [53]. It should be noted that age-related correlations for the radii of curvature for inner lens structures may not be as accurate. By virtue of the age, these regions of the lens will undergo more changes in their structural proteins that can affect optical clarity and localised refractive index than layers closer to the periphery. Therefore, our calculations, which do not consider localised biological changes, may introduce deviations from true values.

### Light scattering

The cross-sectional OCT images of the lens indicate the non-homogenous character of the optical properties of the crystalline lens (Fig. 1c). The OCT signal in the images represents back-scattered light and is associated with optical micro-heterogeneity of the lens i.e., differences in the local refractive index [11, 21, 29]. Far more light is back-scattered from the cortex than from the nucleus where more light is absorbed with age. Quantitative analysis showed that light scattering in the lens increases significantly with age. Earlier studies investigated this effect and detected non-linear dependence of densitometric measurements with respect to age (e.g., exponential or bilinear) [5, 16, 54, 55]. In this study, the scatter plots of optical density are represented by linear relations, which confirms previous OCT studies [31]. Linear fitting enabled a general assessment of change in optical density and facilitated interpretation of trends. With a larger sample size, a more detailed assessment can be undertaken. However, different definitions of optical density have been used in the various studies, and thus a direct comparison of the rates of change cannot be made [5, 16, 31, 44, 54, 55].

We also showed that the highest increase in scattering occurs in the hyper-reflective zone C3, which was also demonstrated in studies using Scheimpflug imaging [2, 56]. Interestingly, the optical density of the C1 zone declined with age, unlike in the C2, C3, and C4 zones. A closer inspection revealed that the correlation of the optical density with age is highly significant in the case of C1α, but not with C1β (Table 2). The outermost zone C1 is the most active zone biologically as it contains the lens epithelium which differentiates into lens fibre cells and most newly formed lens fibres. The optical density depends on the protein concentration and the apparent decline in optical density of C1 with age may indicate a slowing of tissue accrual with age and hence a decrease in protein concentration as fibre cells take longer to form. It could also be caused by a greater stretching of outer layer cells with age as the lens becomes larger and therefore more dispersal of the proteins within these cells.

### Impact of age-related changes on optical quality of the eye and vision performance

The densitometric effects similar to those in the crystalline lens were also observed in other ocular components. Age-related opacification was previously measured in the cornea [57–59] and in the vitreous [60–62]. The changes in light scattering in the lens correlated significantly with light scattering in the vitreous [61].

Back-scattering is used to evaluate the condition of the eye in imaging modalities like OCT. However, it is forward scattering that occurs during light propagation through the entire eye that contributes to the degradation of optical performance of the eye. OSI is a biomarker that gives objective information on the image formed on the retina. The OSI is calculated as the ratio of the peripheral light intensity (between 12 and 20 arc minutes) to the central peak light intensity (1 arc minute) of the retinal image. Intraocular forward scatter degrades the retinal point spread function, consequently increasing the OSI. We found a significant increase in OSI with age although the correlation (R = 0.32) was weaker than that reported in previous studies that had used the OQAS instrument (Fig. 2d) [63, 64]. This suggests that enhanced intraocular scattering in older subjects, which is predominantly from the lens, is a factor responsible for the decrease in optical quality of the eye. In addition, a moderate correlation between OSI (forward scatter) and optical density of the lens extracted from OCT data (back scatter) comes from the fact that forward- and back-scattered light are governed by different physical processes [65], and that OCT and OQAS operate at different wavelengths and different scattering angles. This also explains why age-dependence of OSI may be weaker than that for optical density (Table 3, Additional file 2).

Reduced optical quality of the eye compromises its visual performance. In addition, OSI correlated more strongly with visual performance metrics than did optical density of the lens (see Additional file 2). This could be explained by the fact that OSI measures forward scattering in the eye, and VA and contrast sensitivity are related to the central part of the retinal point spread function.

### Limitations

The main limitation of our study was the size of the participant group. Consequently, we used non-parametric testing of the relations between the extracted parameters and age, which allowed an inference to be made about the general trend with age. In addition, we recruited a single participant from the first decade of life and eight teenagers. Accordingly, a limited sample size and age range did not permit a more thorough investigation of changes in lens thickness, which have been reported in vivo and ex vivo within 20 years after birth [3, 30, 31].

Given our sample size of 49, the power of 80% (α = 0.05) can be achieved for Spearman’s correlation coefficients that is higher or equal to 0.4. This indicates that the study was sufficiently powered to identify moderate and strong (but not weak) correlations. The study proved to be able to determine significant correlations with absolute Spearman’s coefficient values up to 0.86, and *P* values for many of the correlations were < 0.0001. In addition, key results were significant after correction for multiple testing. For the analysis of age-groups, based on analysis of variance, the power was calculated for percent contributions of individual layers in Groups 1, 2, and 3 with sizes 21, 18, and 9 subjects, respectively. The power levels for zones C1α, C1β, C2, C3 and C4 were 43%, 35%, 95%, 98%, and 74%, respectively. The power above 70% meant that most of the correlations with age are high enough to justify the sample size. Larger sample sizes would provide improved correlations of the already highly significant age-related differences in the lens, cortex, and nucleus as well as in OSD zones found here.

The analysis is limited since no mydriasis was applied before scanning, which restricted the field of view in the OCT images. Moreover, the assumed model of the eye used in refraction correction algorithm determined the morphometry of the lens. No gradient refractive indices were considered during data post-processing since we used an equivalent refractive index for the cortex and nucleus for all processed data. The refractive index of the lens alters with age [11–13, 29] which may have influenced the apparent posterior lens curvature. The refraction correction methods equivalent to our approach showed that the accuracy of estimation of posterior radius of curvature of the lens should not be worse than 0.4 mm [66, 67]. However, different solutions are emerging for a robust reconstruction of the shape of the lens using in vivo OCT data [68, 69].

The zones of discontinuity are an interesting physiological feature seen in healthy lenses. The clinical significance is, therefore, not immediately evident given that clinical findings generally are associated with pathological changes. However, with further studies and with advancement in clinical practice and approach, it is anticipated that the nature of these zones and their significance, in clinical applications will become more apparent. Knowledge about the healthy lens is needed to form a basis for diagnosing changes resulting from pathologies and the rate at which these pathologies may progress. The research applications are very promising as it may be possible in the future to determine the growth phases of the lens and to learn more about human lens development and ageing.

## Conclusions

Three-dimensional OCT enabled high-resolution crystalline lens imaging which showed that the cortex contributes more to lens thickness change with age than does the nucleus. The nucleus and cortical C2-C4 zones contribute to the increase in opacification of the lens with age. The most significant age-related changes took place in the C3 zone as it thickens faster and becomes opaquer than the other OSD zones. Changes in the geometry and transparency of the crystalline lens have a significant but weak impact on optical quality of the eye and its visual performance. These insights complement studies on lens structure and the major protein classes and can help towards an improved understanding of the structure/function relationship of the lens.

### Supplementary Information

**Additional file 1.** Intra-observer and inter-observer reproducibility of the thicknesses and optical densities of the optical signal discontinuity zones.

**Additional file 2.** Correlations between the optical density of the lens and the parameters describing optical quality of the eye (objective scatter index, OSI) and visual function (visual acuity, VA, and the area under the log contrast sensitivity function, AULCSF).

## Data Availability

The data generated during and/or analysed during the current study are available in the Repository for Open Data (RepOD), https://doi.org/10.18150/BFGJ2G.

## Abbreviations

OSD: Optical signal discontinuity
OCT: Optical coherence tomography
OQAS: Optical quality analysis system
OSI: Objective scatter index
VAO: Visual adaptive optics simulator
AULCSF: Area under the log contrast sensitivity function
ROI: Region of interest
VA: Visual acuity
ICC: Intraclass correlation coefficient
