# Supplementary material for: Age-related changes in geometry and transparency of human crystalline lens revealed by optical signal discontinuity zones in swept-source OCT images
Source: Eye Vis (Lond). 2023 Dec 1;10:46. doi: 10.1186/s40662-023-00365-y (PMC10691129; doi:10.1186/s40662-023-00365-y)
Supplement: Supplementary file 1 — Additional file 1. Intra-observer and inter-observer reproducibility of the thicknesses and optical densities of the optical signal discontinuity zones. [file 40662_2023_365_MOESM1_ESM.docx]

**Intra-observer and inter-observer reproducibility of the thicknesses and optical densities of the OSD zones**

The OCT data of 10 randomly selected eyes were used to assess the reproducibility of the measurements. Two observers (AG and IG) performed manual segmentation of the lenticular optical signal discontinuity (OSD) zones. The observers used the same criteria for segmentation. The measurements of the thickness and optical density from OCT-derived densitograms were completed using a dedicated automatic software. Three segmentation sessions were completed by each observer, and the order of segmentation was random within the session. The observers were also blinded to their previous measurements and to each other's results. The intra-observer reproducibility was assessed by calculating the intraclass correlation coefficient (ICC) for each parameter (for both observers separately). The inter-observer reproducibility was determined by calculating ICC for mean parameter values. The ICC was calculated using the function *icc* from irr 0.84.1 in R v.4.3.1. The results of reproducibility analysis are presented in the Additional Table 1.

**Additional Table 1.** Intra- and inter-observer reproducibility of optical signal discontinuity (OSD) zone thickness and optical density.

| **Parameter** | **Intra-observer reproducibility** | | | | **Inter-observer reproducibility** | |
| --- | --- | --- | --- | --- | --- | --- |
|  | **Observer 1** | | **Observer 2** | | **ICC (95% CI)** | ***P*** |
|  | **ICC (95% CI)** | ***P*** | **ICC (95% CI)** | ***P*** |  |  |
| **Thickness** |  |  |  |  |  |  |
| Lens | 0.9995  (0.9985–0.9999) | <10−5 | 0.9998  (0.9994–0.9999) | <10−5 | 0.9999  (0.9995–0.9999) | <10−5 |
| Cortex | 0.9755  (0.9326–0.9932) | <10−5 | 0.9842  (0.9560–0.9957) | <10−5 | 0.9884  (0.9569–0.9971) | <10−5 |
| Nucleus | 0.8111  (0.5647–0.9433) | <10−5 | 0.8718  (0.6856–0.9627) | <10−5 | 0.8726  (0.5910–0.9663) | 0.00012 |
| C1α | 0.8907  (0.7265–0.9685) | <10−5 | 0.9702  (0.9186–0.9918) | <10−5 | 0.9046  (0.6817–0.9750) | <10−5 |
| C1β | 0.8578  (0.6565–0.9584) | <10−5 | 0.9639  (0.9020–0.9900) | <10−5 | 0.6249  (0.0682–0.8899) | 0.0159 |
| C2 | 0.8902  (0.7255–0.9684) | <10−5 | 0.9375  (0.8353–0.9824) | <10−5 | 0.8213  (0.4591–0.9517) | 0.0006 |
| C3 | 0.9788  (0.9415–0.9942) | <10−5 | 0.9624  (0.8980–0.9896) | <10−5 | 0.9611  (0.8604–0.9900) | <10−5 |
| C4 | 0.3799  (0.0002–0.7582) | 0.0249 | 0.6640  (0.3248–0.8906) | 0.00016 | 0.3103  (–0.3309–  0.7656) | 0.1658 |
| **Optical density** |  |  |  |  |  |  |
| Lens | 0.9972  (0.9921–0.9992) | <10−5 | 0.9998  (0.9995–0.9999) | <10−5 | 0.9971  (0.9889–0.9993) | <10−5 |
| Cortex | 0.9904  (0.9732–0.9974) | <10−5 | 0.9980  (0.9942–0.9994) | <10−5 | 0.9793  (0.9240–0.9947) | <10−5 |
| Nucleus | 0.9926  (0.9792–0.9980) | <10−5 | 0.9998  (0.9995–0.9999) | <10−5 | 0.9919  (0.9699–0.9980) | <10−5 |
| C1α | 0.9940  (0.9830–0.9984) | <10−5 | 0.9956  (0.9877–0.9988) | <10−5 | 0.9351  (0.7752–0.9832) | <10−5 |
| C1β | 0.9898  (0.9715–0.9972) | <10−5 | 0.9938  (0.9824–0.9983) | <10−5 | 0.8478  (0.5252–0.9593) | 0.0003 |
| C2 | 0.9883  (0.9672–0.9968) | <10−5 | 0.9936  (0.9820–0.9983) | <10−5 | 0.9753  (0.9099–0.9937) | <10−5 |
| C3 | 0.9989  (0.9968–0.9997) | <10−5 | 0.9963  (0.9894–0.9990) | <10−5 | 0.9952  (0.9822–0.9988) | <10−5 |
| C4 | 0.9766  (0.9355–0.9935) | <10−5 | 0.9339  (0.8266–0.9814) | <10−5 | 0.9523  (0.8311–0.9878) | <10−5 |

ICC = intraclass correlation coefficient; CI = confidence interval

The analysis showed that all parameters except for the measurement of the thickness of zone C4 are highly reproducible.
