# Supplementary material for: Age-related changes in geometry and transparency of human crystalline lens revealed by optical signal discontinuity zones in swept-source OCT images
Source: Eye Vis (Lond). 2023 Dec 1;10:46. doi: 10.1186/s40662-023-00365-y (PMC10691129; doi:10.1186/s40662-023-00365-y)

**Additional file 2**. Correlations between the optical density of the lens and the parameters describing optical quality of the eye (OSI) and visual function [visual acuity (VA), and the area under the log contrast sensitivity function (AULCSF)].


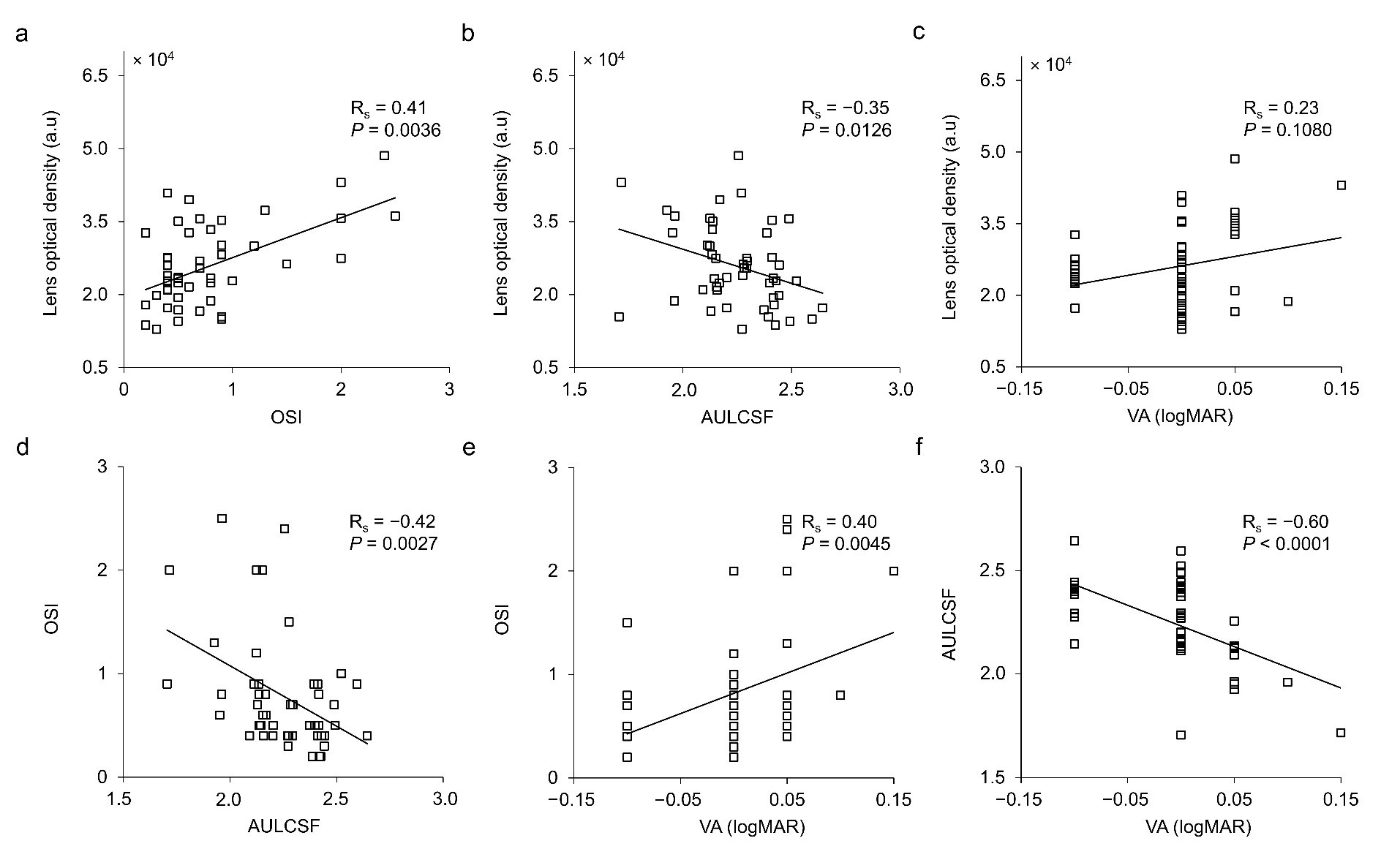

Supplement: Supplementary file 2 — Additional file 2. Correlations between the optical density of the lens and the parameters describing optical quality of the eye (objective scatter index, OSI) and visual function (visual acuity, VA, and the area under the log contrast sensitivity function, AULCSF). [file 40662_2023_365_MOESM2_ESM.docx]
